# Visualization of multivalent histone modification in a single cell reveals highly concerted epigenetic changes on differentiation of embryonic stem cells

**DOI:** 10.1093/nar/gkt528

**Published:** 2013-06-12

**Authors:** Naoko Hattori, Tohru Niwa, Kana Kimura, Kristian Helin, Toshikazu Ushijima

**Affiliations:** ^1^Division of Epigenomics, National Cancer Center Research Institute, 5-1-1 Tsukiji, Chuo-ku, Tokyo 104-0045, Japan, ^2^Biotech Research and Innovation Centre (BRIC), University of Copenhagen, Ole Maaløes Vej 5, 2200 Copenhagen, Denmark and ^3^Centre for Epigenetics, University of Copenhagen, Ole Maaløes Vej 5, 2200 Copenhagen, Denmark

## Abstract

Combinations of histone modifications have significant biological roles, such as maintenance of pluripotency and cancer development, but cannot be analyzed at the single cell level. Here, we visualized a combination of histone modifications by applying the *in situ* proximity ligation assay, which detects two proteins in close vicinity (∼30 nm). The specificity of the method [designated as imaging of a combination of histone modifications (iChmo)] was confirmed by positive signals from H3K4me3/acetylated H3K9, H3K4me3/RNA polymerase II and H3K9me3/H4K20me3, and negative signals from H3K4me3/H3K9me3. Bivalent modification was clearly visualized by iChmo in wild-type embryonic stem cells (ESCs) known to have it, whereas rarely in *Suz12* knockout ESCs and mouse embryonic fibroblasts known to have little of it. iChmo was applied to analysis of epigenetic and phenotypic changes of heterogeneous cell population, namely, ESCs at an early stage of differentiation, and this revealed that the bivalent modification disappeared in a highly concerted manner, whereas phenotypic differentiation proceeded with large variations among cells. Also, using this method, we were able to visualize a combination of repressive histone marks in tissue samples. The application of iChmo to samples with heterogeneous cell population and tissue samples is expected to clarify unknown biological and pathological significance of various combinations of epigenetic modifications.

## INTRODUCTION

Histone modifications are known to play important roles in various biological and pathological processes, such as cell type-specific gene expression and cancer development (1,2). In addition, the crucial importance of their combinations is indicated by recent findings, such as the presence of cell type-specific multivalent histone modifications revealed by genome-wide analyses (3–7) and proteins that recognize a combination of histone modifications (8). Especially, the combination of histone H3 lysine 4 trimethylation (H3K4me3) and histone H3 lysine 27 trimethylation (H3K27me3), named the bivalent modification, is present almost exclusively in pluripotent stem cells, such as embryonic stem cells (ESCs) (3,5), early stage embryos (9) and immature T-cells (10), and is thought to maintain the ‘stemness’ of these cells. Furthermore, aberrant expression of a protein that binds to a specific combination of histone modifications was associated with poor prognosis in breast cancer, suggesting the importance of a combination also in pathological processes (11).

Regardless of the crucial importance of combinations of histone modifications, methodologies to detect combinations are so far limited to the sequential-chromatin immunoprecipitation (sequential-ChIP) assay (3,12) and recently developed immunoprecipitation-mass spectrometry assay (13). Although a sequential-ChIP assay has the advantage to identify genomic regions with a specific combination of histone modifications, it suffers from the necessity of a large number of cells, and its application is limited to samples containing >10^6^ cells. Moreover, in samples with heterogeneous cell populations, it is impossible to identify cells with a specific combination of histone modifications. Owing to this limitation, we cannot identify which cells have a specific multivalent modification in samples derived from tissues, and even in a cell line if the sample consists of cells at various differentiation stages.

In this study, we aimed to visualize the coexistence of two histone modifications by applying the *in situ* proximity ligation assay (*in situ* PLA), an imaging technique of protein–protein interactions (14). Based on the principle of *in situ* PLA, if two different modifications recognized by respective first antibodies exist approximately within 30 nm, oligonucleotide probes conjugated to their secondary antibodies can serve as a template for rolling-circle amplification. The amplification products can hybridize with fluorescent probes and be detected as fluorescence signals, reflecting the combination of histone modifications. In addition, we applied the method to analyze the presence of a specific combination of histone modifications in heterogeneous cell populations and tissue sections.

## MATERIALS AND METHODS

### Culture of mouse ESCs and embryonic fibroblasts

J1 ESC line and *Suz12* knockout (KO) ESC line (clone SBE8) established as reported (15) were cultured in normal ESC medium with 15% fetal bovine serum and 1000 U/ml of leukemia inhibitory factor (LIF) (ESGRO, Chemicon, CA) on mitotically inactivated mouse embryonic fibroblasts (MEFs) kindly provided by Dr Shiota K. (The University of Tokyo). For immunofluorescence staining and imaging of a combination of histone modifications (iChmo), MEFs at passage three were purchased from Millipore (Billerica, MA) and cultured in Dulbecco’s modified Eagle’s medium with 10% fetal bovine serum for 6 days at passage five. To induce differentiation of ESCs, cells were seeded on 0.1% gelatin-coated cell plate at a density of 3.0 × 10^5^ cells/100 mm dish in the absence of feeder layer cells and LIF and then pre-cultured for 1 day. After pre-culture, ESCs were cultured for 24 or 48 h with 1 µM of all-trans retinoic acid (RA) (Sigma, St. Louis, MO). The medium was changed every day.

### Preparation of mouse and human tissue samples

For RNA extraction, mouse liver and brain were resected from 8-week-old C57BL/6J male mice purchased from CLEA Japan, Inc. (Tokyo, Japan). For preparation of histological sections, human colonic tissues resected with a colon cancer were obtained, embedded in Tissue-Tek O.C.T. Compound (Sakura Finetek Japan, Tokyo, Japan) and frozen on dry ice. Sections of 4 µm of thickness were prepared for immunofluorescence staining and iChmo without a dry step. The animal experiments were approved by the Committee for Ethics in Animal Experimentation at the National Cancer Center. Human samples were obtained with informed consent, and the analysis was approved by the institutional review boards.

### Immunofluorescence staining

For immunofluorescence staining, cells and tissue sections were fixed with 4% formaldehyde for 15 min, washed three times in PBS and permeabilized by 1% Triton X-100 in PBS for 20 min. After washing five times in PBS, cells and sections were incubated in blocking buffer (1% BSA in PBS) for 30 min and then with mouse monoclonal antibodies directed against H3K4me3 (1:1000; Wako, Tokyo, Japan; 307-34813), H4K20me3 (1:1000; Abcam, Cambridge, MA; ab78517), Oct-3/4 (1:500; Santa Cruz Biotechnology, Santa Cruz, CA; sc-5279) or ßIII-tubulin (1:500; Covance, Berkeley, CA; MMS-435P), or with rabbit polyclonal antibodies directed against acetylated H3K9 (H3K9ac) (1:500, Abcam; ab10812), H3K9me3 (1:1000, Millipore: 07-442), H3K27me3 (1:1000, Millipore; 07-449) or RNA polymerase II (RNAPII) (1:500, Abcam; ab5095) in the same buffer for 1 h. The specificity of antibodies against histone modifications was confirmed previously (16,17). Cells and sections were washed in PBS three times for 5 min and incubated in Alexa Fluor 594-conjugated goat anti-rabbit IgG (1:1000, Invitrogen, Carlsbad, CA) or Alexa Fluor 488-conjugated goat anti-mouse IgG (1:1000, Invitrogen) for 1 h. After washing with PBS three times for 5 min, coverslips were mounted using ProLong Gold antifade reagent with 4′,6-diamidino-2-phenylindole (DAPI) (Invitrogen). Fluorescence of cultured cells stained with histone modification antibodies was detected under a laser-scanning confocal microscope (LSM710; Carl Zeiss, Oberkochen, Germany), and all images were acquired and analyzed using LSM Software ZEN 2008 (Carl Zeiss). Images of Oct-4 and ßIII-tubulin staining of ESCs and of human tissue sections were captured using a FV10iW laser-scanning confocal microscope (Olympus, Tokyo, Japan).

### iChmo

Duolink *in situ* PLA was purchased from Olink Bioscience (Uppsala, Sweden). First, cells and tissue sections were fixed, permeabilized and incubated with primary antibodies under the same condition in the immunofluorescence staining. The primary antibodies were the same as those for the immunofluorescence staining, and the concentrations were two times higher than those for the immunofluorescence staining. Then, samples were incubated with secondary antibodies conjugated with PLA probes MINUS and PLUS at 37°C for 90 min. Finally, the PLA probes MINUS and PLUS were ligated using two connecting oligonucleotides to produce a template for rolling-cycle amplification. After amplification, the amplification products were hybridized with red fluorescence-labeled oligonucleotide. Samples were mounted on coverslips using ProLong Gold antifade reagent with DAPI. Fluorescence of cultured cells was detected under LSM710, and all images were acquired and analyzed using ZEN 2008. For quantitative analysis of iChmo signals, the fluorescence spots were counted using Z-stack acquisition of BZ-9000 microscope system. Fluorescence of tissue sections was captured using a FV10iW laser-scanning confocal microscope.

### Quantitative reverse transcription-PCR

DNase-treated total RNA (1 µg) was reverse-transcribed with Oligo-dT_20_ (Invitrogen, Carlsbad, CA) and Superscript III reverse transcriptase (Invitrogen). Quantitative PCR (qPCR) was carried out by real-time PCR using SYBR® Green I. The primer sequences and PCR conditions are shown in Supplementary Table S1. The amplification curve of a sample was compared with those of standard DNA samples with known copy numbers to obtain the copy number in the sample. The number of target cDNA molecules was normalized to those of mouse *Gapdh* cDNA molecules.

### Statistical analysis

To evaluate significant difference between two independent groups of sample data, the Mann–Whitney *U*-test was used.

## RESULTS

### Combinations of epigenetic marks are visualized by iChmo

**Figure 1.**
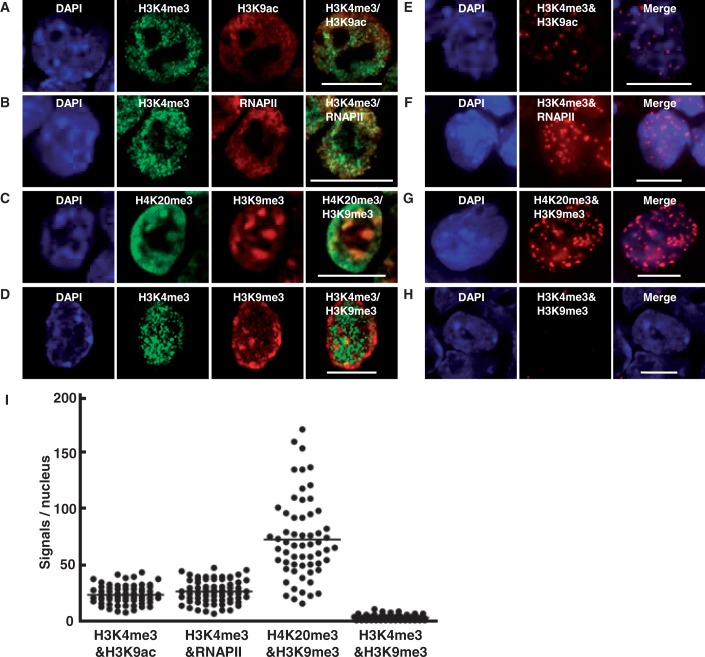
Visualization of combinations of epigenetic modifications in a single cell. Immunofluorescence staining was performed using mouse ESCs and antibodies against H3K4me3 and H3K9ac (**A**), H3K4me3 and RNAPII (**B**), H4K20me3 and H3K9me3 (**C**) and H3K4me3 and H3K9me3 (**D**). Colocalizations of H3K4me3/H3K9Ac, of H3K4me3/RNAP2 and of H4K20me3/H3K9me3 were observed, whereas that of H3K4me3/H3K9me3 was not. iChmo was performed using mouse ESCs and antibodies against H3K4me3 and H3K9ac (**E**), H3K4me3 and RNAPII (**F**), H4K20me3 and H3K9me3 (**G**) and H3K4me3 and H3K9me3 (**H**). Coexistence of H3K4me3/H3K9ac, of H3K4me3/RNAPII and of H4K20me3/H3K9me3, but not of H3K4me3/H3K9me3, was observed. Scale bar represents 10 µm. (**I**) The number of iChmo spots was counted for individual combinations in the nuclei of ESCs (H3K4me3 and H3K9ac, *n* = 71; H3K4me3 and RNAPII, *n* = 70; H4K20me3 and H3K9me3, *n* = 60; and H3K4me3 and H3K9me3, *n* = 80).

To visualize a combination of histone modifications, we first performed the *in situ* PLA focusing on two combinations of epigenetic marks known to be present at active loci, one H3K4me3 and H3K9ac and the other H3K4me3 and RNAPII (18). Immunofluorescence staining of mouse ESCs confirmed that the signals of H3K4me3 and H3K9ac, and those of H3K4me3 and RNAPII, were observed as merged signals in the nucleus (Figure 1A and B). The *in situ* PLA for the combinations of H3K4me3/H3K9ac and H3K4me3/RNAPII demonstrated a large number of fluorescence spots in the nucleus (Figure 1E and F). The possibility that the signals were derived from non-specific binding of PLA probes was excluded by the absence of fluorescence spots of the *in situ* PLA using antibodies against H3K4me3 and H3K9me3 that were not merged by immunofluorescence (Figure 1D and H). Quantitatively, the mean number of spots of H3K4me3/H3K9ac, H3K4me3/RNAPII and H3K4me3/H3K9me3 was 22.9, 25.9 and 2.4 per nucleus, respectively (Figure 1I). Therefore, the coexistence of epigenetic marks was visualized at the single cell level by applying the *in situ* PLA, and this method was designated as iChmo.

Next, we analyzed whether iChmo can also detect a combination of two marks at a different histone protein of the same or neighboring nucleosomes within 30 nm, such as H3 and H4. By immunofluorescence using antibodies against H3K9me3 and H4K20me3, epigenetic marks for heterochromatin, their colocalization was confirmed by the presence of merged signals (Figure 1C). By iChmo using the same two antibodies, a large number of spots were produced (Figure 1G), and the mean number of spots was 72.5 per nucleus (Figure 1I), indicating that iChmo can visualize a combination of epigenetic marks, even if they are at different histone proteins in a close proximity.

### Bivalent modification is specifically visualized at the single cell level

**Figure 2.**
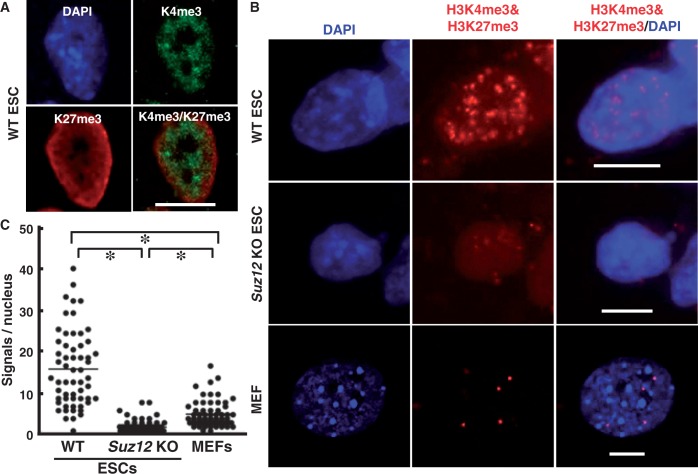
Application of iChmo to visualization of bivalent modification. (**A**) Mouse ESCs were stained by immunofluorescence with antibodies against H3K4me3 and H3K27me3 (scale bar: 10 µm). (**B**) Coexistence of H3K4me3 and H3K27me3 in WT ESCs was detected by iChmo, but hardly in *Suz12* KO ESCs and MEFs (scale bar: 10 µm). (**C**) The mean number of fluorescence spots was significantly larger in WT ESCs (15.2; *n* = 58) than in *Suz12* KO ESCs (0.9; *n* = 69) and MEFs (4.1; *n* = 60) (Mann–Whitney *U*-test; *P < 0.001).

We focused on bivalent modification because of its biological significance and applied iChmo to visualize it using mouse ESCs and MEFs that have a lot of and few, respectively, bivalent modifications (3,5). By immunofluorescence staining of ESCs, H3K4me3 signals were observed as interspersed small dots, whereas H3K27me3 signals were enriched at the periphery of the nuclear membrane (Figure 2A). Some signals were merged in the nucleus, appearing to reflect the presence of bivalent modification. However, the same staining pattern was observed in MEFs (Supplementary Figure S1), and it was shown that immunofluorescence was not capable of distinguishing whether H3K4me3 and H3K27me3 were in close proximity. However, notably, by iChmo, a large number of fluorescence spots were observed in ESCs, whereas only a small number of spots were in MEFs (Figure 2B). This demonstrated that iChmo can distinguish whether two modifications coexist in the vicinity at the single cell level.

### iChmo signals of bivalent modification are decreased in MEFs and Suz12 KO ESCs

To further confirm that iChmo signals originated from bivalent modification, we performed iChmo using *Suz12* KO ESCs. Suz12 is a component of Polycomb repressive complexes 2 together with Ezh2 and Eed (19), and is required for Polycomb repressive complexes 2 enzymatic activity (20). In the *Suz12* KO ESCs, global loss of H3K27me3 was observed by western blotting (15) and by immunofluorescence (Supplementary Figure S1), and loss of genomic regions with bivalent modification by quantitative ChIP-PCR (Supplementary Figure S2). iChmo produced no or few fluorescence spots of bivalent modification in the nucleus of *Suz12* KO ESCs (Figure 2B). The fact that this technique itself worked even in *Suz12* KO ESCs and MEFs was confirmed by detecting the coexistence of H3K4me3/H3K9ac and H3K4me3/RNAPII in these cells (Supplementary Figure S3). Quantitatively, the mean number of spots from bivalent modification was 0.9 and 4.1 per nucleus in the *Suz12* KO ESCs (69 nuclei counted) and MEFs (60 nuclei counted), respectively, whereas it was 15.2 in wild-type (WT) ESCs (58 nuclei counted) (Figure 2C). These data clearly showed that application of iChmo enabled us to visualize the bivalent modifications at the single cell level.

### iChmo reveals highly concerted epigenetic changes during ESC differentiation

**Figure 3.**
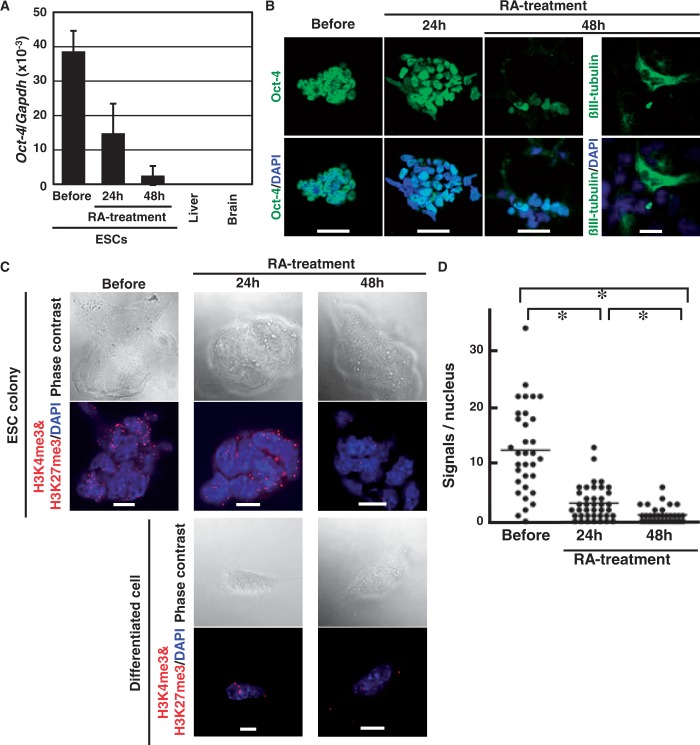
Visualization of epigenetic and phenotypic layers of differentiation in an early stage of ESC differentiation. (**A**) Differentiation of ESCs was induced by treatment of all-trans RA, and *Oct-4* mRNA expression was measured before, and 24 and 48 h after the RA treatment. The expression levels in the mouse liver and brain are shown as those in *Oct-4*-negative tissues. Values show mean + SD of three experiments. (**B**) Expression of Oct-4 and ßIII-tubulin proteins was analyzed in ESCs before, and 24 and 48 h after the RA treatment by immunofluorescence (scale bar: 20 µm). (**C**) Images of phase contrast and iChmo for the bivalent modification in ESC colonies before, and 24 and 48 h after the RA treatment (upper panel), and differentiated neuron-like cells at 24 and 48 h (lower panel). Regardless of the phenotypic differentiation statuses, the bivalent modification was absent both at 24 and 48 h, supporting highly concerted regulation of epigenetic changes. Scale bar represents 10 µm. (**D**) The number of fluorescence spots was counted in ESCs before (*n* = 33), and 24 (*n* = 37) and 48 (*n* = 38) h after the RA treatment (Mann–Whitney *U*-test; *P < 0.001). Although the decrease of Oct-4 expression and increase of ßIII-tubulin were highly variable among the ESCs treated with RA, the decrease of the bivalent modification was highly coordinated.

We took advantage of an imaging method to analyze individual cells in a heterogeneous sample, namely, ESCs at an early stage of differentiation. ESC differentiation was induced by all-trans RA treatment 24 h after removal of LIF and feeder layer cells. The expression of *Oct-4* mRNA decreased to the half 24 h after the RA treatment, but was still detectable 48 h after the treatment (Figure 3A). At cellular level, Oct-4 protein was detectable in all ESCs before the treatment but was heterogeneously detectable 24 h later (Figure 3B). At 48 h, in accordance with the residual *Oct-4* mRNA expression, a minor fraction of ESCs still had the Oct-4 protein expression (Figure 3B). ßIII-tubulin, a differentiated neuron marker, was also expressed in only a fraction of the ESCs (Figure 3B), showing the presence of large variations in the phenotypic differentiation of the ESCs. In contrast, no or little fluorescence spots of the bivalent modification were observed 24 and 48 h after the RA treatment by iChmo, regardless of whether ESCs formed colonies or were differentiated into neuron-like cells (Figure 3C). The mean number of spots of ESCs at 48 h after the RA treatment was at the same level as that of the *Suz12* KO ESCs (Figure 3D). These data indicated that the epigenetic layer of differentiation was completed at 2 days in a highly concerted manner, whereas the phenotypic layer of differentiation proceeded with considerable variation.

### iChmo visualizes a combination of histone modifications in tissue samples

**Figure 4.**
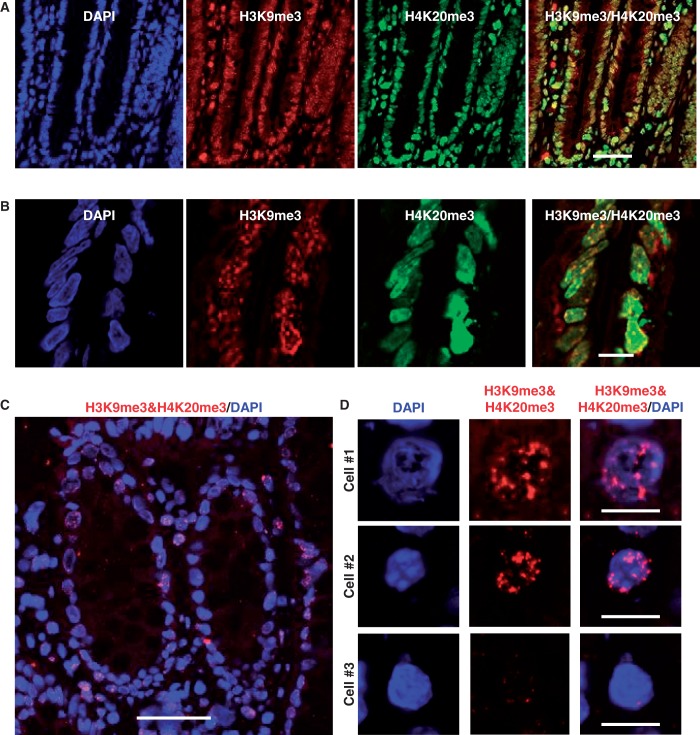
Application of iChmo to the analysis of human colonic tissue. (**A**) Human colonic tissues were stained by immunofluorescence with antibodies against H3K9me3 and H4K20me3 (scale bar: 50 µm). Colocalization of H3K9me3/ H4K20me3 was observed in the cells of colonic tissue. (**B**) High-magnification images of (A) (scale bar: 10 µm). (**C**) Coexistence of H3K9me3 and H4K20me3 was visualized in the nuclei of cells as fluorescence spots (scale bar: 50 µm). (**D**) High-magnification images of (C) (scale bar: 10 µm). The cells with iChmo spots (Cell #1 and #2) coincided with the cells having weak DAPI intensity, and the cells without iChmo spots (Cell #3) showed strong DAPI intensity.

If histone combinations in individual cells can be analyzed in tissues, this will enable us to identify cell populations of unique functions, such as stem cells. To apply iChmo to a human tissue sample, we first screened 11 antibodies that had high specificity applicable to iChmo using cultured cells and identified that a combination of antibodies against H3K9me3 and H4K20me3 could be used for tissues samples (Figure 4A and B). Small dots of H3K9me3 were detected in all the cells of a human colonic tissue, presenting a similar staining pattern to that of cultured cells. Signals of H4K20me3 were also detected, and small dots of H3K9me3 were merged with those of H4K20me3 (Figure 4B). By iChmo, fluorescence spots were observed (Figure 4C) and were localized in the nuclei of cells (Figure 4D). Notably, the appearance of iChmo spots of H3K9me3 and H4K20me3 showed heterogeneity among the cells, and the cells that lacked iChmo spots corresponded to those that had strong DAPI intensity (Figure 4C and Supplementary Figure S4A and B), indicating that the presence of a combination of H3K9me3 and H4K20me3 was dependent on a cell condition, such as the cell cycle. The possibility that fluorescence spots were produced by non-specific binding of PLA probes was excluded by the absence of iChmo spots (Supplementary Figure S4E) using antibodies against H3K9ac and H4K20me3 (Supplementary Figure S4C and D).

## DISCUSSION

An imaging method for a combination of epigenetic modifications in close proximity was established and was designated as iChmo. It had the capacity to visualize the combinations at the single cell level and thus was able to analyze heterogeneous samples, such as a cell line consisting of cells at various differentiation stages and tissue samples.

The application of iChmo to ESCs at their differentiation revealed that the epigenetic layer of differentiation takes place in a highly concerted manner before the phenotypic layer of differentiation. Previous reports showed that gene expression changes at several genes occur in parallel with histone modification changes during ESC differentiation (21,22). In addition, it is reported that, at *Hoxa1* and *Sox21*, development-related genes marked with bivalent modification, H3K27me3 levels decreased before gene activation at the early stage of ESC differentiation (23,24). However, up to now, it has been impossible to analyze to what degree such histone modification changes are concerted among cells during ESC differentiation, and the use of iChmo revealed highly concerted change. Also, taking advantage of its applicability to a heterogeneous cell population and the role of bivalent modification in ‘stemness’, iChmo might be able to evaluate the level of cell reprogramming and the quality of iPS cells (25).

iChmo is also used for analysis of tissue sections. Although we analyzed only a limited number of combinations of histone modifications for now owing to availability of specific antibodies, analysis of tissue samples is expected to produce rich information, such as localization of tissue stem cells. Indeed, Lien *et al.* (4) clearly showed that the combination of H3K4me3 and H3K79me2 was specifically found in quiescent hair follicle stem cells by ChIP-sequencing. We can expect that, by using iChmo, the presence of such combination of histone modifications can be demonstrated in a small amount of cells of histological sections. Also, aberrant expression of histone modifiers is now becoming evident in human disorders, especially in cancers (26), and iChmo might clarify which aberrant combinations are present, even if the fraction of cells with such aberrant combinations is small.

Analysis of human colonic tissue samples revealed the presence of heterogeneity of iChmo spots of H3K9me3 and H4K20me3, and the cells without iChmo spots coincided with those with strong DAPI intensity (Supplementary Figure S4). It is known that DAPI intensity is affected by DNA content, reflecting the phase of the cell cycle. In addition, considering that the levels of H3K9me3 and H4K20me3 dynamically change during the cell cycle (27), we can speculate that the heterogeneity of iChmo spots of H3K9me3 and H4K20me3 reflects the difference of the phases of the cell cycle.

The maximum distance between the primary antibodies recognized by two PLA probes has been estimated to be ∼30 nm (14), which is longer than the minimum distance between two nucleosomes (∼15 nm). Thus, there remains a possibility that combinations of histone modifications visualized in this study originated from two neighboring nucleosomes. However, the fact that few fluorescence spots were produced for the combination of H3K4me3 and H3K27me3 in MEFs supported that the signals are produced from two modifications at a close distance, theoretically within two neighboring nucleosomes.

iChmo showed a large number of spots of bivalent modification in ESCs, but only a small number of spots in MEFs, which was in line with a report by Bernstein *et al.* (3). At the same time, Mikkelsen *et al.* (5) reported that a half of bivalent marks in mouse ESCs still remained in MEFs. The apparent discrepancy between the iChmo data and the previous report can be accounted for by two possibilities; (i) because one iChmo spot does not always reflect one combination of histone modifications owing to the principle of *in situ* PLA (14), and all regions with coexistence of histone modifications are not visualized by this method, and (ii) because the number of bivalent marks is different according to the culture period of MEFs. In this study, we used MEFs cultured at passage five, whereas Mikkelsen *et al.* (5) used primary MEFs, and the authors also suggested the latter possibility.

To summarize, a specific combination of histone modifications was visualized by applying the *in situ* PLA, and the method was capable of analyzing heterogeneous cell population and tissue samples. Application of the method has the potential to uncover previously unknown biological and pathological significances of combinations of histone modifications.

## SUPPLEMENTARY DATA

Supplementary Data are available at NAR Online: Supplementary Table 1, Supplementary Figures 1–4, Supplementary Methods and Supplementary Reference [28].

## FUNDING

Grant-in-Aid for the Third-Term Comprehensive Cancer Control Strategy from the Ministry of Health, Labour and Welfare, Japan (to T.U.); A3 Foresight Program from the Japan Society for the Promotion of Science (to T.U.); Research Grants in the Natural Sciences of The Mitsubishi Foundation (to T.U. and N.H.). Funding for open access charge: Third-Term Comprehensive Cancer Control Strategy from the Ministry of Health, Labour and Welfare, Japan.

*Conflict of interest statement*. None declared.

## Supplementary Material

Supplementary Data
