# Supplementary material for: Visualization of multivalent histone modification in a single cell reveals highly concerted epigenetic changes on differentiation of embryonic stem cells
Source: Nucleic Acids Res. 2013 Jun 12;41(15):7231–9. doi: 10.1093/nar/gkt528 (PMC3753646; doi:10.1093/nar/gkt528)
Supplement: Supplementary Data [file supp_41_15_7231__index.html]

Visualization of multivalent histone modification in a single cell reveals highly concerted epigenetic changes on differentiation of embryonic stem cells — Visualization of multivalent histone modification in a single cell reveals highly concerted epigenetic changes on differentiation of embryonic stem cells — Supplementary Data 

# Visualization of multivalent histone modification in a single cell reveals highly concerted epigenetic changes on differentiation of embryonic stem cells

## 

files

**Files in this Data Supplement:**

- Supplementary Data - pdf file
