# Supplementary material for: Visualization of multivalent histone modification in a single cell reveals highly concerted epigenetic changes on differentiation of embryonic stem cells
Source: Nucleic Acids Res. 2013 Jun 12;41(15):7231–9. doi: 10.1093/nar/gkt528 (PMC3753646; doi:10.1093/nar/gkt528)
Supplement: Supplementary Data [file supp_gkt528_nar-03218-x-2012-File006.pdf]

SUPPLEMENTARY MATERIAL

Visualization of Multivalent Histone Modification in a Single Cell Reveals Highly Concerted Epigenetic Changes upon Differentiation of Embryonic Stem Cells

Naoko Hattori, Tohru Niwa, Kana Kimura, Kristian Helin, Toshikazu Ushijima

Supplementary Data

Supplementary Table S1, Supplementary Figure S1, S2, S3, and S4

Supplementary Methods

Supplementary References

Supplementary Table S1. Primers for ChIP-PCR and RT-PCR

	Gene symbol	Sequence (forward)	Sequence (reverse)	Anealing temp. (˚C)
**ChIP-PCR**	*Piwil1*	GTGTCCACCTCGCCGTTA	ACCTGCTAACAGCCCCAACT	62
	Il13	CCTCACTCTGGCCTTCTCTG	TTGGAGCTGAAAGAGGGAAA	58
	Sox9	TATTAGAGACCCTGAGCTGGAAGT	CTGGACTGAAACTGGTAAAGTTGT	58
	Brachury	CTTTGTTTCTTCCCGCTGAG	GCAAACCTGGTCATTCCAGT	58
	Sox21	GGCGGACAGAAACACAAACA	GCAAGATTCTCCAGGCAGGT	58
	Ppia	CGTTTGGAAAGCAGTTGTGA	GACAGTGGCGTCTGCAAAG	58
	Actb	AAATGCTGCACTGTGCGGCG	AGGCAACTTTCGGAACGGCG	58
**RT-PCR**	*Pou5f1* (*Oct-4*)	AGATCACTCACATCGCCAAT	GGTGTCCCTGTAGCCTCATA	57
	Gapdh	CTGAACGGGAAGCTCACTGG	ATGCCTGCTTCACCACCTTC	54

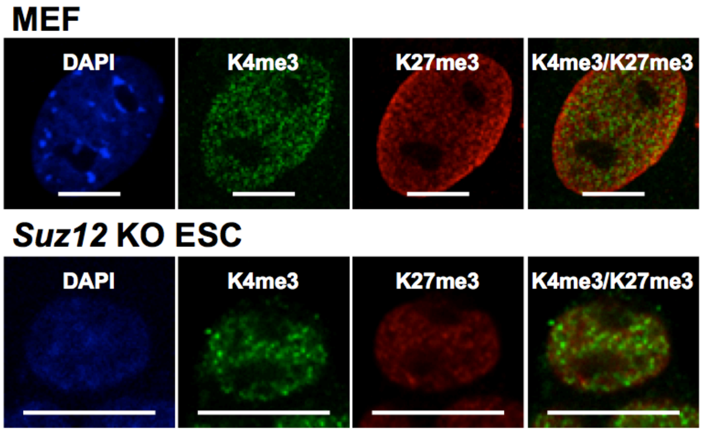

Supplementary Figure S1. Immunofluorescence staining of MEFs and *Suz12* KO ESCs

MEFs and *Suz12* KO ESCs were stained by immunofluorescence using antibodies against H3K4me3 and H3K27me3. Scale bar: 10 μm. The colocalization pattern of H3K4me3 and H3K27me3 in MEFs was indistinguishable from that of ESCs. In *Suz12* KO ESCs, H3K27me3 signals were lost, and colocalization was not observed.

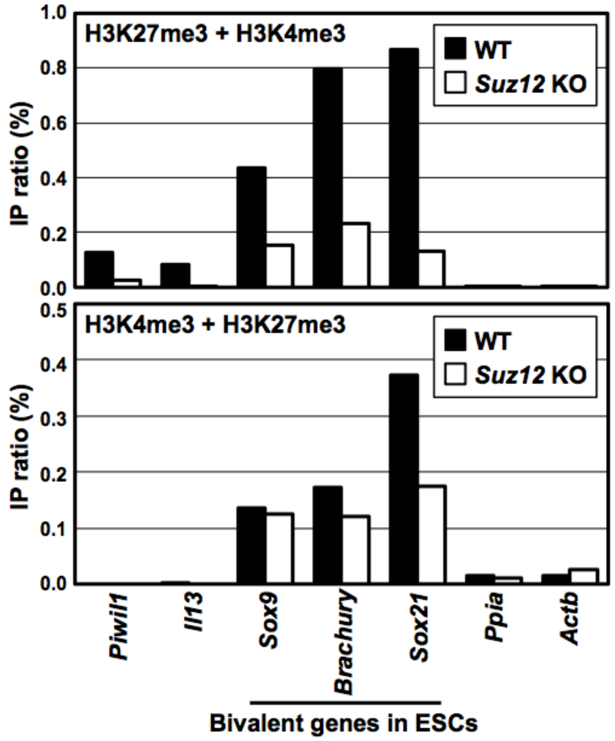

Supplementary Figure S2. Loss of H3K27me3 at genes with bivalent modifications in *Suz12* KO ESCs

Sequential ChIP was performed to examine the presence of bivalent modifications at two genes only with H3K27me3 (*Piwil1* and *Il13*), three genes with bivalent modifications (*Sox9*, *Brachury*, and *Sox21*), and two genes only with H3K4me3 (*Ppia* and *Actb*). The IP ratio was analyzed by quantitative PCR. (Upper panel) Using the antibody against H3K27me3 first and then that against H3K4me3, the bivalent modification was not detected for *Piwil1*, *Il13*, *Ppia*, and *Actb*. Amounts of the bivalent modification at *Sox9*, *Brachury*, and *Sox21* markedly decreased in the *Suz12* KO ESCs. (Lower panel) Using the antibody against H3K4me3 first and then that against H3K27me3, similar results were obtained.

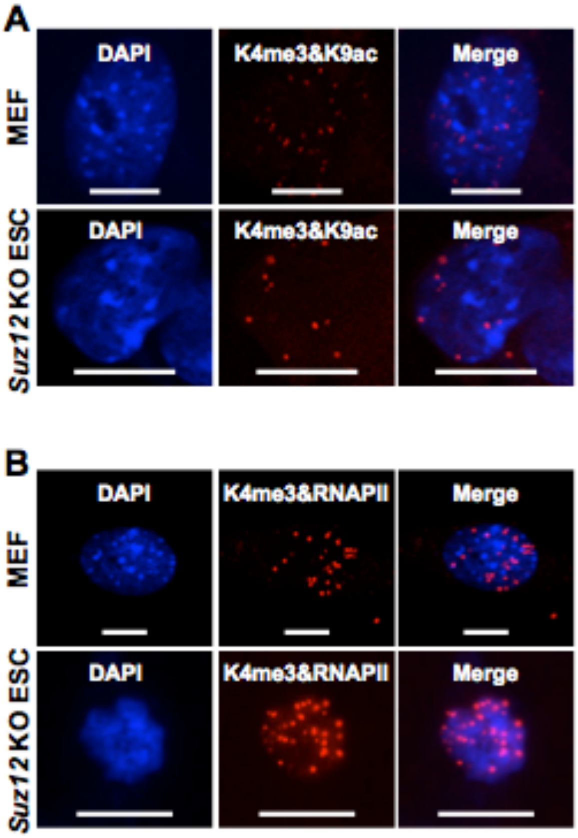

Supplementary Figure S3. Coexistence of H3K4me3 and RNAPII in MEFs and *Suz12* KO ESCs

(A and B) The iChmo visualized coexistence of H3K4me3 and H3K9Ac (A), and of H3K4me3 and RNAPII (B) both in MEFs and *Suz12* KO ESCs. This showed that the iChmo itself worked in these cells. Scale bar represents 10 μm.

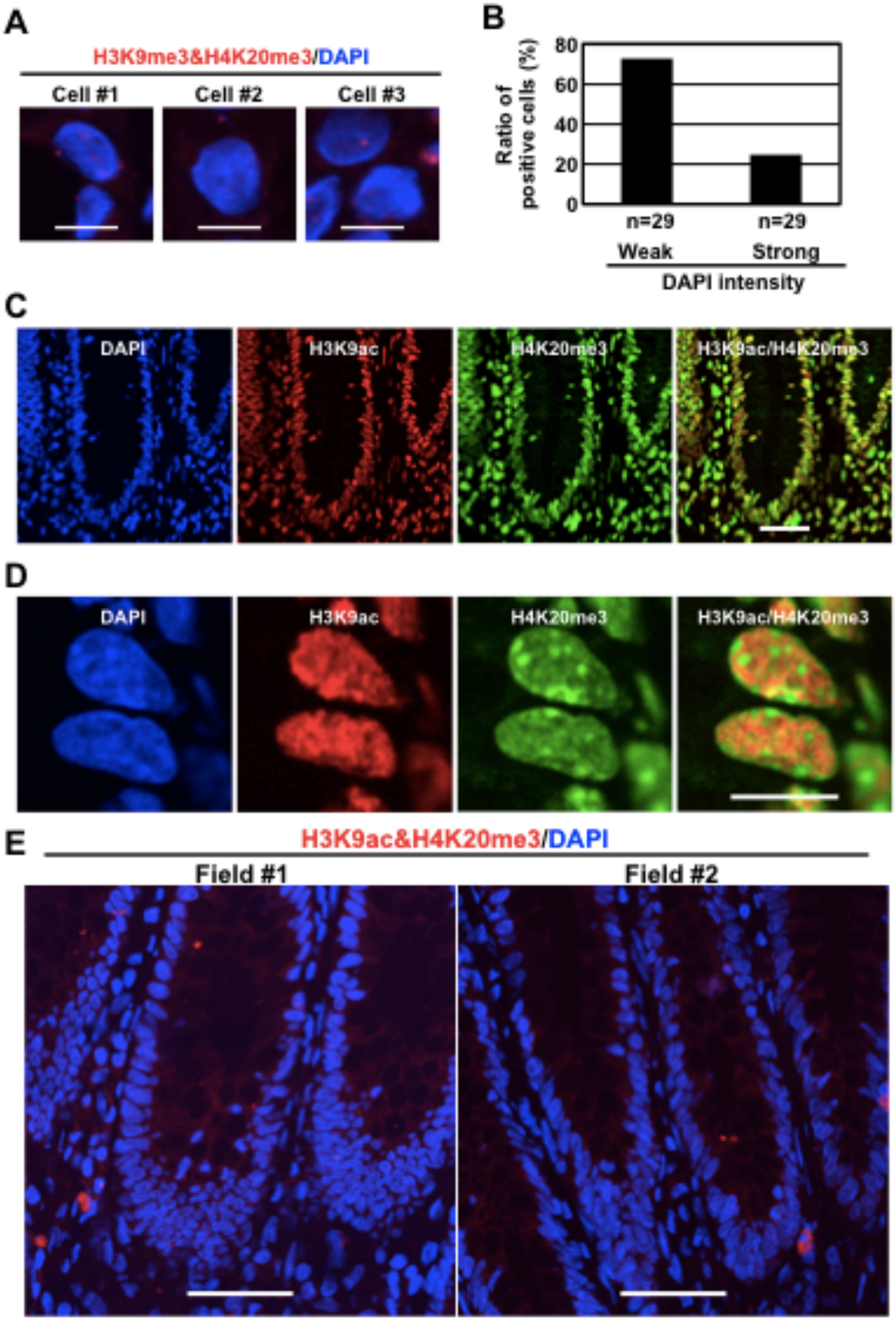
A
H3K9me3&H4K20me3/DAPI
Cell #1
Cell #2
Cell #3
B
Ratio of positive cells (%)
80
60
40
20
0
n=29
n=29
Weak
Strong
DAPI intensity
C
DAPI
H3K9ac
H4K20me3
H3K9ac/H4K20me3
D
DAPI
H3K9ac
H4K20me3
H3K9ac/H4K20me3
E
H3K9ac&H4K20me3/DAPI
Field #1
Field #2

Supplementary Figure S4. Application of iChmo to the analysis of human colonic tissues

(A) Representative images of the cells with strong DAPI intensity (#1, #2, and #3) that did not show iChmo spots of H3K9me3 and H4K20me3 in human colonic crypts (scale bar: 10 μm). (B) Quantitatively, 72.4% of the cells with weak DAPI intensity and 24.1% of the cells with strong intensity showed iChmo spots, showing the association between the presence of iChmo spots and DAPI intensity. (C) Human colonic tissues were stained by immunofluorescence with antibodies against H3K9ac and H4K20me3 (scale bar: 50 μm). (D) High-magnification images of (C) (scale bar: 10 μm). Fluorescence signals of H3K9ac (red) and H4K20me3 (green) were essentially mutually exclusive. (E) No iChmo spots were observed using antibodies against H3K9ac and H4K20me3 in human colon tissues (scale bar: 50 μm).

Supplementary Methods

Sequential-chromatin immunoprecipitation assay

Before harvest, J1 and *Suz12* KO ESCs were cultured on dishes twice for 30 min to exclude contamination of feeder layer cells. Cross-link, sonication, and a first immunoprecipitation with antibody against H3K27me3 (Millipore; 07-449) or against H3K4me3 (Abcam; ab8580) were carried out as described (Takeshima et al., 2009, Genome Res.). After the first immunoprecipitation, chromatin was eluted in a solution of 30 mM DTT, 500 mM NaCl, and 0.1% SDS at 37˚C for 30 min. Eluted chromatin was diluted 50-fold, subjected to a second immunoprecipitation with antibody against H3K4me3 (Abcam; ab8580) or against H3K27me3 (Millipore; 07-449), and then eluted with TE buffer. Cross-link in the eluate was reversed in the presence of 200 mM NaCl overnight at 65˚C. The quantitative ChIP-PCR was performed using the isolated DNA as described previously (Takeshima et al., 2009, Genome Res.). The primer sequences and PCR conditions are shown in Supplementary Table 1.

Supplementary Reference

Takeshima, H., Yamashita, S., Shimazu, T., Niwa, T. and Ushijima, T. (2009) The presence of RNA polymerase II, active or stalled, predicts epigenetic fate of promoter CpG islands. *Genome Res.*, 19, 1974-1982.
